# Developmental Localization and Methylesterification of Pectin Epitopes during Somatic Embryogenesis of Banana (*Musa* spp. AAA)

**DOI:** 10.1371/journal.pone.0022992

**Published:** 2011-08-03

**Authors:** Chunxiang Xu, Lu Zhao, Xiao Pan, Jozef Šamaj

**Affiliations:** 1 College of Horticulture, South China Agricultural University, Guangzhou, Guangdong, China; 2 Department of Cell Biology, Centre of the Region Haná for Biotechnological and Agricultural Research, Faculty of Science, Palacký University, Olomouc, Czech Republic; 3 Institute of Plant Genetics and Biotechnology, Slovak Academy of Sciences, Nitra, Slovak Republic; Iowa State University, United States of America

## Abstract

**Background:**

The plant cell walls play an important role in somatic embryogenesis and plant development. Pectins are major chemical components of primary cell walls while homogalacturonan (HG) is the most abundant pectin polysaccharide. Developmental regulation of HG methyl-esterification degree is important for cell adhesion, division and expansion, and in general for proper organ and plant development.

**Methodology/Principal Findings:**

Developmental localization of pectic homogalacturonan (HG) epitopes and the (1→4)-β-D-galactan epitope of rhamnogalacturonan I (RG-I) and degree of pectin methyl-esterification (DM) were studied during somatic embryogenesis of banana (*Musa* spp. AAA). Histological analysis documented all major developmental stages including embryogenic cells (ECs), pre-globular, globular, pear-shaped and cotyledonary somatic embryos. Histochemical staining of extracellularly secreted pectins with ruthenium red showed the most intense staining at the surface of pre-globular, globular and pear-shaped somatic embryos. Biochemical analysis revealed developmental regulation of galacturonic acid content and DM in diverse embryogenic stages. Immunodots and immunolabeling on tissue sections revealed developmental regulation of highly methyl-esterified HG epitopes recognized by JIM7 and LM20 antibodies during somatic embryogenesis. Cell walls of pre-globular/globular and late-stage embryos contained both low methyl-esterified HG epitopes as well as partially and highly methyl-esterified ones. Extracellular matrix which covered surface of early developing embryos contained pectin epitopes recognized by 2F4, LM18, JIM5, JIM7 and LM5 antibodies. De-esterification of cell wall pectins by NaOH caused a decrease or an elimination of immunolabeling in the case of highly methyl-esterified HG epitopes. However, immunolabeling of some low methyl-esterified epitopes appeared stronger after this base treatment.

**Conclusions/Significance:**

These data suggest that both low- and highly-methyl-esterified HG epitopes are developmentally regulated in diverse embryogenic stages during somatic embryogenesis. This study provides new information about pectin composition, HG methyl-esterification and developmental localization of pectin epitopes during somatic embryogenesis of banana.

## Introduction

The production of banana (*Musa* spp.), one of the most important fruit crops in the world, is seriously threatened by cold stress and pests such as *Fusarium oxysporum* var. *cubense*. Development of new banana cultivars with resistance to diseases or cold stress is one of the best ways to overcome this problem. However, it is extremely difficult to create such cultivars through conventional breeding because most commercial cultivars are sterile triploids bearing parthenocarpic fruits. Plant regeneration *via* somatic embryogenesis is the base of banana germplasm improvement using biotechnological techniques. Unfortunately, some important banana cultivars are recalcitrant in regard to the embryogenic response [1]–[4]. Solution of this problem represents a major challenge for future studies aiming at improvement of the banana germplasm.

Cell wall plays a very important role in the plant development. The chemical components of the cell walls are modulated during plant growth and development. Several previous studies have reported about developmental changes in cell wall components such as arabinogalactan proteins and pectins in some plant species such as maize (*Zea mays* L.), chicory (*Cichorium*), coconut (*Cocos nucifera* L.), barley (*Hordeum vulgare*) and olive (*Olea europaea* L.) [5]–[10]. However, to our knowledge, no study was devoted to cell wall pectins during somatic embryogenesis of banana.

The walls of plant cells are primarily composed of cellulose, hemicellulose (e.g., xyloglucans, xylans, and mannans), pectins, and a small amount of structural proteins. Pectins, one major class of chemical components, make up to 35% of the primary cell walls in dicotyledonous plants and non-graminaceous (non-grass) monocots [11]. Homogalacturonan (HG) is the most abundant pectin polysaccharide, making up to 65% of total pectin [12]. The structural domains of pectin are built on more or less methyl- and acetyl-esterified galacturonan. One main characteristic of pectin is the extent of methyl-esterification on the carboxyl group of polygalacturonic acid. The degree of HG methyl-esterification has been reported as the key determinant of plant and organ development involving processes such as cell division, expansion, and adhesion [12], [13]. Furthermore, a minimum stretch of nine unmethylated galacturonic acid (GalA) residues can form Ca^2+^ linkages, which may promote the formation of so-called “egg-box” model structure [14]. Hence, the methyl-esterification status of HG can have dramatic consequences on cell wall texture and mechanical properties, thereby contributing to cell shape and growth [12].

Somatic embryogenesis is characterized by well-defined embryogenic stages, which are generally similar to those in zygotic embryogenesis. This process requires strict spatial and temporal control over cell division and elongation [15]. In some plants, digestion of cell wall pectins by pectinase can result in complete or partial disappearance of the extracellular matrix (ECM) at the surface of embryogenic cells (ECs) and/or proembryos, thus leading to their collapse [16], [17]. These observations point to the importance of pectins for somatic embryogenesis and ECM structural integrity.

Currently, immunohistochemical techniques using well characterized antibodies have been applied to better define plant cell wall components and to localize cell wall polymers *in situ* within complex tissues and organs. These techniques enable monitoring of structural changes, organization and partial changes of function in the plant cell wall [18]. Indeed, the application of immunohistochemical technique by using monoclonal antibodies JIM5 and JIM7 led to the identification of pectic epitopes in the extracellular matrix surface network (ECMSN) of calli in chicory [7] and kiwifruit (*Actinidia deliciosa*) [17]. The same epitopes were also detected during organogenesis from callus of wheat (*Triticum aestivum* L.) [19], and also during microspore embryogenesis of *Citrus*, cork oak (*Quercus suber* L.), olive and *Capsicum annuum* L. [10], [20]–[22]. With the help of the 2F4 antibody, Liners et al. [23] monitored the distribution of pectic polysaccharides in cell walls of carrot (*Daucus carota* L.) suspension cells and sugar beet (*Beta vulgaris* L.) calli. Another immunohistochemical study described the changes of JIM5 and JIM7 epitopes during somatic embryogenesis of coconut [9].

Ruthenium red is a cationic stain with six positive charges, which forms electrostatic bonds to the acidic groups of sugars, for example carboxyl groups and sulfate groups [24]. Usually, it is used to study mucilage secreted by plant seeds. Mucilage is composed of complex acidic or neutral polysaccharide polymers of high molecular weight, mostly methyl-esterified HG [25], rhamnogalacturonan I, and low amount of galactans and arabinans [26]. Ruthenium red was also successfully used to study amount of galacturonic acid (GalA) in carrot callus [27].

In this study, ruthenium red along with an arsenal of pectin antibodies, including six recently developed antibodies specifically recognizing HG epitopes with varying degrees of methyl-esterification [28], [29], were used to map the developmental distribution and regulation of these epitopes during banana somatic embryogenesis. Moreover, the two above mentioned independent localization techniques were complemented by biochemical and immunodot analyzes.

## Results

### Histological examination of somatic embryogenesis in banana

**Figure 1:**
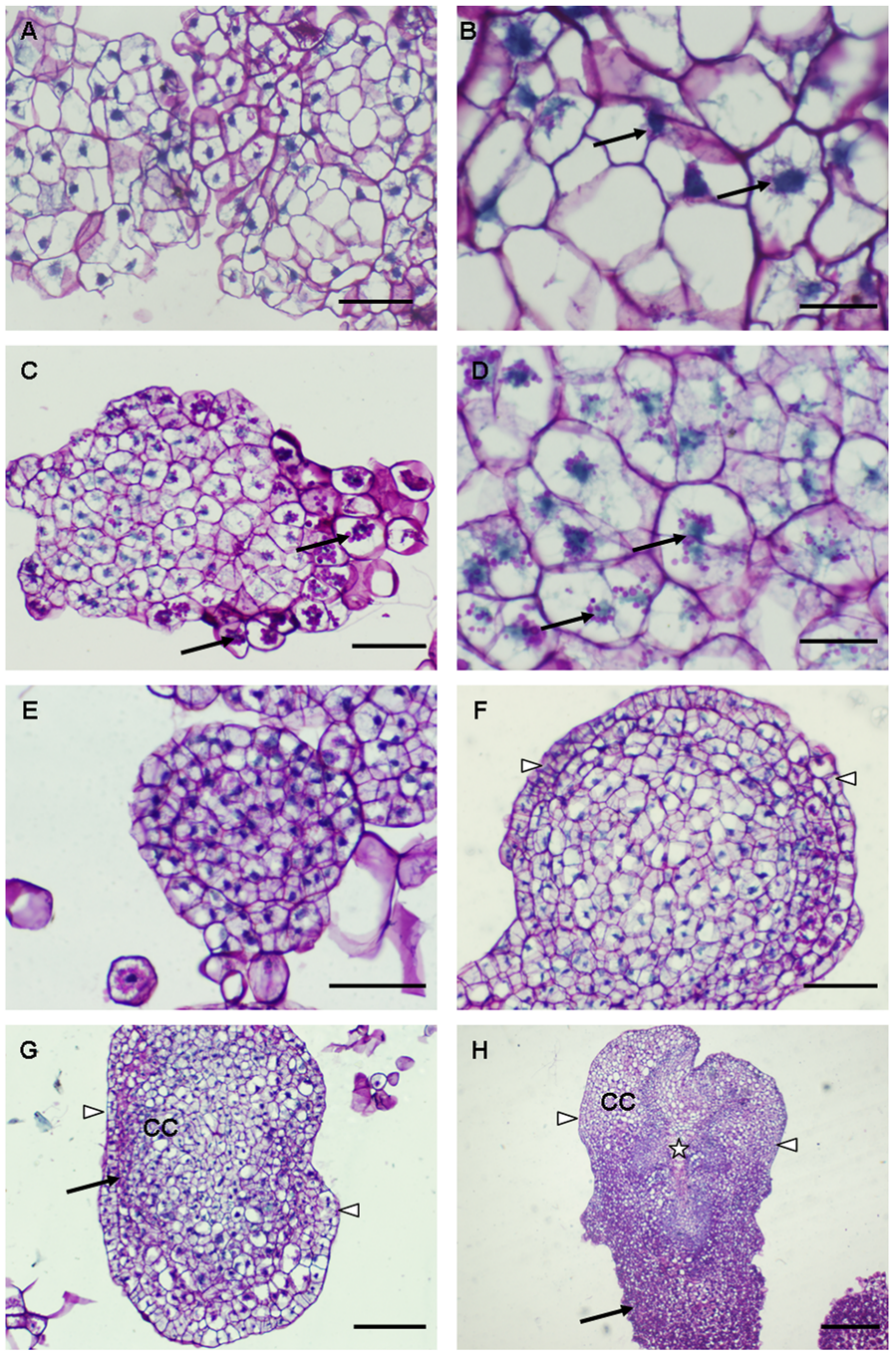
Histological analysis of NECs in banana cultivar ‘Baxijiao’ (A, B) and ECs and somatic embryos in cultivar ‘Yueyoukang 1’ after periodic acid-Schiff reagent (PAS) staining. Starch and surface mucilage were stained pink while nuclei were stained blue. A. An overview of NECs; B. A detail of NECs showing very few starch granules (pink staining, arrows) around nuclei (blue staining). C. An overview of ECs partially surrounded by “starch-rich cells” (arrows); D. A detail of ECs showing many starch granules (pink staining, arrows), especially around nuclei (blue staining); E. A median histological section through pre-globular embryo; F. A median histological section through globular embryo with well-developed epidermis (arrowheads); G. A median histological section through pear-shaped embryo showing epidermis (arrowheads), subepidermal and cortical cells (CC) with starch (arrow); H. A median histological section through cotyledonary embryo showing epidermis (arrowheads), cortical cells (CC), starch-rich cells in the basal (arrow) as well as in the lateral parts of the embryo and vascular tissue in the middle part of the embryo (star); Bars represent 20 µm in B and D; 50 µm in A, C, E and F; 100 µm in G and 200 µm in H.

Previously, PAS has been used to detect glycogen in animal tissues and carbohydrates, proteoglycans and glycoproteins in plant tissues. It is based on the reaction of periodic acid which selectively oxidizes the glucose residues and creates aldehydes reacting with the Schiff reagent (product of fuchsine or pararosaniline and sodium bisulfite) and producing purple-magenta color. In the present study, PAS staining was used to study the histology of NECs of cultivar ‘Baxijiao’ used as a control as well as in ECs and somatic embryos of cultivar ‘Yueyoukang 1’. This histological analysis revealed different cellular organization in NECs versus ECs as well as all basic developmental embryogenic stages including ECs, pre-globular, globular, pear- shaped and cotyledonary somatic embryos of cultivar ‘Yueyoukang 1’ (Fig. 1). In more detail, very few small starch granules were found in the cytoplasm of NECs, especially around their nuclei (Fig. 1A, B). In comparison to NECs, ECs were smaller but they contained more and relatively bigger starch granules localized around their nuclei (Fig. 1C, D). Small ECs were surrounded by some bigger cells, which were strongly stained by PAS and contained the biggest starch granules (Fig. 1C). Therefore, these cells were termed “starch-rich cells” in this study. Starch content decreased in pre-globular and globular embryos (Fig. 1E, F) while many starch granules re-appeared in the cells localized in lateral and basal parts of late-stage pear-shaped and cotyledonary embryos (Fig. 1G, H). The formation of vascular tissue was initiated in cotyledonary embryos (Fig. 1H). PAS staining revealed mucilage-like areas and layers, especially at the surface of NEC and EC clumps (Fig. 1A, B) as well as at the outer surface of pre-globular, globular and pear-shaped embryos (Fig. 1E–G) and parts of cotyledonary embryos containing starch-rich cells (Fig. 1H).

### Histochemical staining of surface-localized pectins with ruthenium red

**Figure 2:**
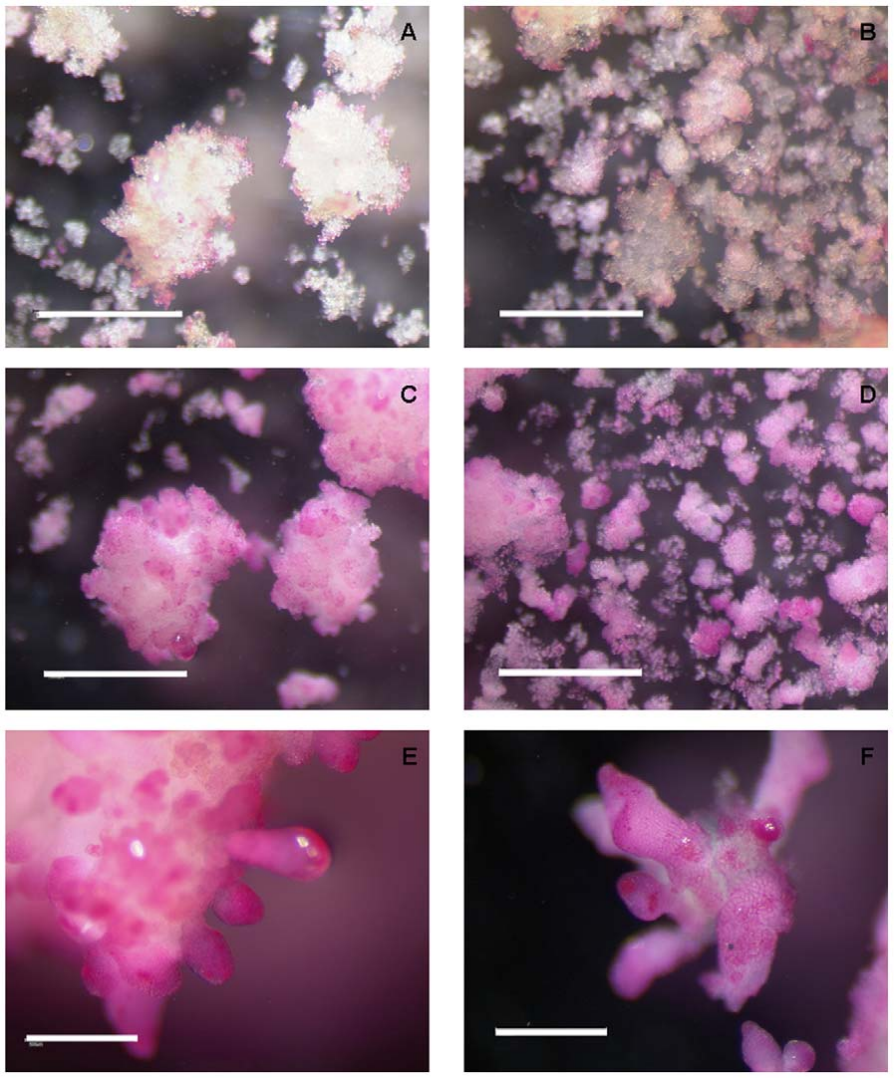
Whole-mount histochemical staining of surface-localized pectins in banana NECs of cultivar ‘Baxijiao’ (A, B) as well as in ECs and somatic embryos of cultivar ‘Yueyoukang 1’ (C–F) with ruthenium red. A. and B. Histochemical staining of clumps composed of NECs. Please, note very weak pink labeling only in few peripherally localized cells and small cell groups; C. and D. Histochemical staining of clumps composed of ECs. Please, note stronger pink/red staining of EC clusters; E. Cluster of embryos at different embryogenic stages from pre-globular to globular and pear-shaped. Please, note an intense pink/red staining at the surface of these somatic embryos. F. Cluster of late-stage cotyledonary embryos. Please, note weaker staining of these embryos and a net-like structure at their outer surface; Bars represent 1 mm in A–D, and 500 µm in E and F.

A surface treatment of the whole mounted fresh material (not fixed and not embedded and sectioned) with ruthenium red was used to label extracellularly secreted pectins in the mucilage covering both non-embryogenic and embryogenic cell clumps as well as somatic embryos. Overall, the intensity of pink/red stain on NECs of cv. ‘Baxijiao’ (Fig. 2A, B) was much weaker than on ECs and somatic embryos of cv. ‘Yueyoukang 1’ (Fig. 2C–F). In more detail, only few surface localized NECs and their clumps were stained by ruthenium red (Fig. 2A, B). In contrast, many more ECs and cell clumps were stained with ruthenium red and the pink/red color was much stronger in these ECs as compared to NECs (Fig. 2C, D). Further, outer surface of somatic embryos at pre-globular, globular and pear-shaped stages was the most strongly stained by ruthenium red (Fig. 2E). The pink/red color of ruthenium red became weaker at the surface of late stage cotyledonary embryos (Fig. 2F). Moreover, staining appeared as a net-like structure at the surface of these cotyledonary embryos (Fig. 2F).

### Immunodot analysis of pectins in nonembryogenic cells and during somatic embryogenesis of banana

**Figure 3:**
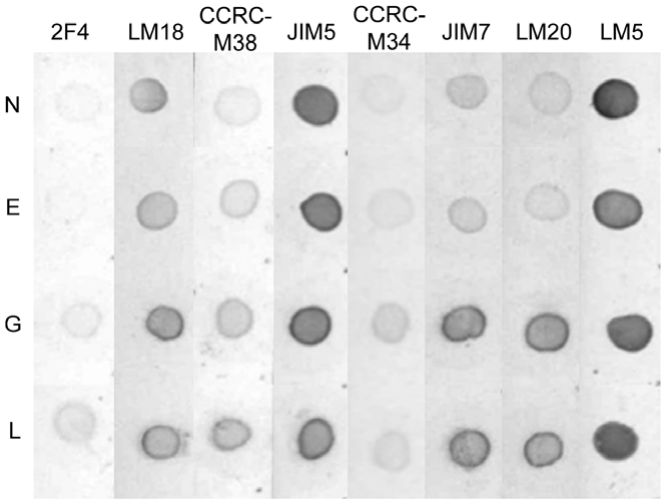
Semi-quantitative developmental analysis by immunodot of the relative abundance of pectin epitopes in extracts prepared from NECs (cultivar ‘Baxijiao’) and from ECs and somatic embryos (cultivar ‘Yueyoukang 1’) during somatic embryogenesis of banana (*Musa* spp. AAA). N: non-embryogenic cells; E: embryogenic cells; P: proembryos; G: globular embryos; L: late embryos.

A representative set of six commercially available monoclonal antibodies (CCRC-M34, CCRC-M38, JIM5, JIM7, LM18 and LM20) has been reported to bind to HG epitopes showing different degrees of esterification. The fully de-esterified and low methyl-esterified HG were recognized by CCRC-M38, JIM5, LM18 and partially to highly methylesterified HG were recognized by JIM7, LM20 and CCRC-M34. These antibodies were tested and selected to study developmental distribution and methyl-esterification of these pectic epitopes in the cell walls of NECs, ECs and somatic embryos of banana by immunodot technique. Furthermore, the 2F4 antibody binding to de-esterified/calcium cross-linked HG and by LM5 antibody mainly binding to (1→4)-β-D-galactan of rhamnogalacturonan I were also used (RG-I) (Table 1, Fig. 3). Additionally, negligible signals were found by using the LM7 (recognizing non-blockwise deesterified HG) and the LM19 antibodies (binding to methyl-esterified HG) in all developmental stages (Table 1, data not shown). This immunodot analysis revealed some semi-quantitative differences in the abundance of diverse pectic epitopes in NECs showing higher abundance of LM5 and JIM5 epitopes, followed by lower abundance of the LM18 epitope (Fig. 3). The abundance of other HG epitopes such as 2F4, CCRC-M34, CCRC-M38, JIM7 and LM20 was relatively low in NECs. The abundance of pectic epitopes in ECs was similar to that of NECs with exception of the 2F4 and LM5 epitopes. The 2F4 epitope was almost undetectable and the LM5 epitope was less abundant in ECs in comparison to NECs (Fig. 3). These differences might be related either to non-embryogenic versus embryogenic character of cell cultures or to the different cultivars used in this study (cultivar ‘Baxijiao’ for NECs versus cultivar ‘Yueyoukang 1’ for ECs). In pre-globular/globular embryos, a relative abundance of highly methyl-esterified HG recognized by LM20, JIM7 and CCRC-M34 antibodies was obviously higher in comparison to ECs (Fig. 3). In late stage cotyledonary embryos, pattern of pectin epitopes resembled previous developmental stage of pre-globular/globular embryos except for a decrease in the abundance of the CCRC-M34 epitope (Fig. 3). These data suggested that several pectin epitopes were changed during progression of somatic embryogenesis in cv. ‘Yueyoukang 1’ (Fig. 3C–F).

**Table 1:** Antibodies used to study distribution of the pectic epitopes in the cell walls of banana embryogenic cultures.

Antibody	Antigen/Epitope	Reference
LM7	Non-blockwise de-esterified HG	26
2F4	De-esterified HG epitope: calcium cross-linked HG	14
CCRCM38	Fully de-esterified HG	29
JIM5	Partially methyl-esterified HG epitope: unesterified residues (up to 40%) adjacent to or flanked by residues with methylester groups	46, 48
LM18	Low methyl-esterified HG	28
LM19	Low methyl-esterified HG	28
JIM7	Partially methyl-esterified HG epitope: methyl-esterified residues up to 80%	46, 48
LM20	Highly methyl-esterified HG	29
CCRCM34	Partially methyl-esterified HG	29
LM5	(1→4)-β-D-galactan	47

### Distribution and methyl-esterification of pectin epitopes in NECs

**Figure 4:**
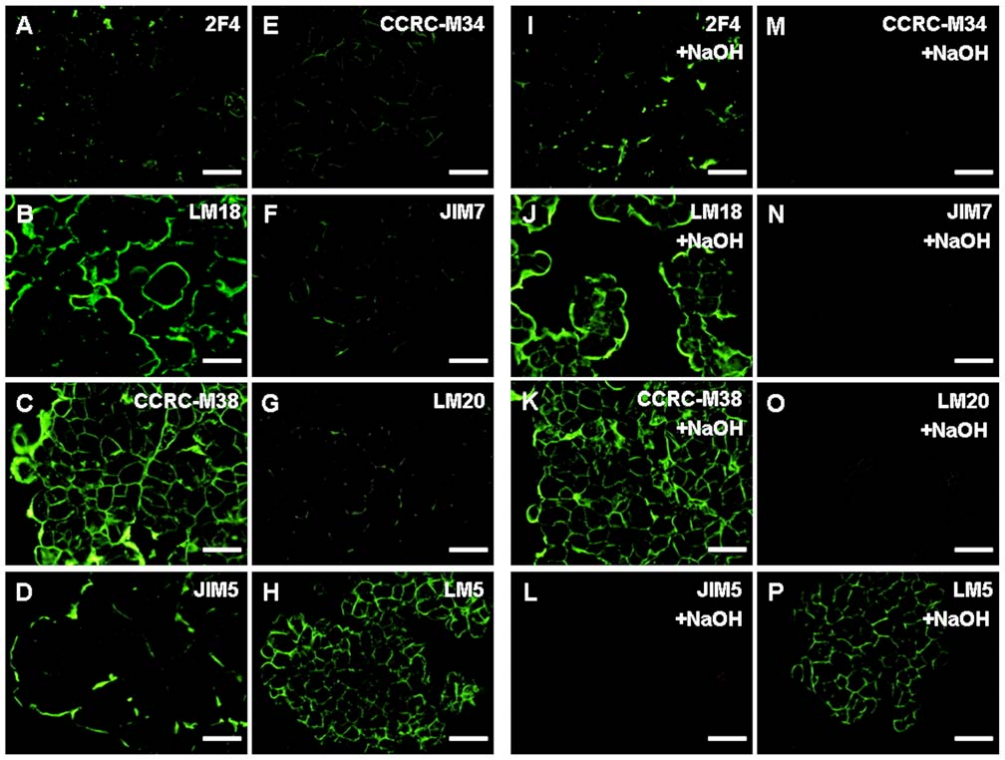
Immunofluorescence localization of pectin epitopes in non-embryogenic cells (NECs) of banana (*Musa* spp. AAA, cultivar ‘Baxijiao’) and the effect of NaOH de-esterification on the immunofluorescence labeling of these epitopes. A and I. The 2F4 epitope was immunolocalized only in few cell walls (A), while base treatment increased this immunolocalization (I); B and J. The LM18 epitope was immunolocalized to the surface of some small cell groups (B) and this immunolabeling slightly increased after base treatment (J); C and K. The CCRC-M38 epitope was immunolocalized all over the section through NECs (C) with no obvious changes after base treatment (K); D and L. The JIM5 epitope was mainly immunolocalized to the surface of some small cell groups (D) but this JIM5 immunolabeling disappeared after base treatment (L); E and M. The CCRC-M34 epitope was weakly immunolocalized in NECs (E) but immunolabeling disappeared after base treatment (M); F and N. The JIM7 epitope showing a very weak immunolocalization in NECs (F) and no visible immunolabeling after base treatment (N); G and O. The LM20 epitope showing a very weak immunolocalization in NECs (G) but no visible immunolabeling after base treatment (O); H and P. The LM5 epitope showing immunolocalization in NECs (H) and slight decrease of immunolabeling after base treatment (P). Bars represent 50 µm.

The immunofluorescence labeling was used to study the presence of diverse pectic epitopes in cell walls. Possible steric hinderance was considered for pectic epitopes during *in situ* immunolabeling due to the possible molecular crowding and/or masking/blocking in intact cell walls. Therefore, both qualitative and quantitative statements about relative abundance of pectic epitopes (as visualized by immunofluorescence labeling) were avoided. Nevertheless, in situ immunolabeling revealed that low and/or fully de-esterified HG epitopes such as LM18, CCRC-M38 and JIM5 as well as in the LM5 epitope representing (1→4)-β-D-galactan (Fig. 4B–D, H) were present in NECs of cultivar ‘Baxijiao’. In contrast, the 2F4 epitope of HG (de-esterified/calcium ion cross-linked HG) was detected only in few cell walls (Fig. 4A). When spatial distribution was taken into account, CCRC-M38 and LM5 epitopes were distributed all over the section in the cell walls of both cortical and inner cell types (Fig. 4C, H) while the LM18 and JIM5 epitopes were located in the surface mucilage areas and layers covering clumps of NECs (Fig. 4B, D). On the other hand, the highly methyl-esterified pectic epitopes CCRC-M34, JIM7 and LM20 were poorly detected in the intact cell walls of NECs (Fig. 4E–G). Next, we tested distribution patterns of these pectic epitopes after chemical de-esterification with NaOH. After such base treatment, immunolabelings of the JIM5-, JIM7- and LM20 epitopes fully disappeared (Fig. 4L, N, O) while they were slightly stronger for the 2F4 (Fig. 4I) and the LM18 (Fig. 4J) epitopes. Further, immunolabeling of the LM5 epitope slightly decreased (Fig. 4P) but no obvious changes were detected for the CCRC-M38 epitope after the base treatment (Fig. 4K).

### Distribution and methyl-esterification of pectin epitopes in ECs

**Figure 5:**
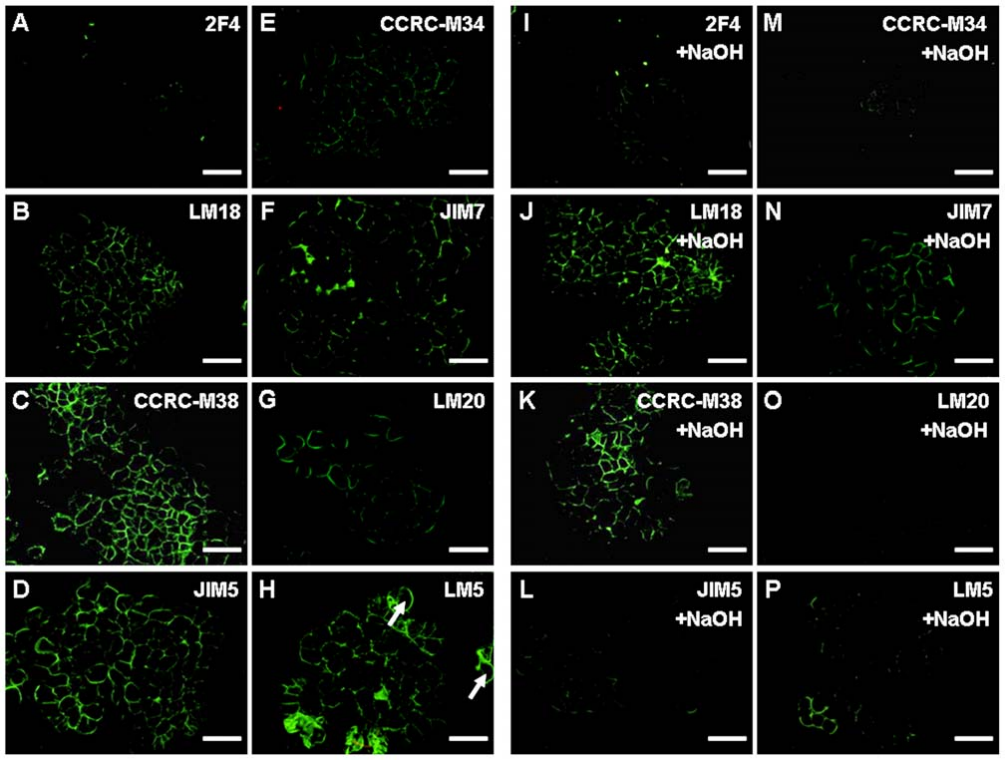
Immunofluorescence localization of pectin epitopes in embryogenic cells (ECs) of banana (*Musa* spp. AAA, cultivar ‘Yueyoukang 1’) and the effect of NaOH de-esterification on the immunofluorescence labeling of these epitopes. A and I. The 2F4 epitope was almost non-dectable by immunolabeling in banana ECs before (A) and also after base treatment (I); B and J. The LM18 epitope was immunolocalizated in ECs (B) with slight increase of immunolabeling after base treatment (J); C and K. The CCRC-M38 epitope showing strong, evenly distributed immunolocalization in ECs (C), with no obvious changes after base treatment (K); D and L. The JIM5 epitope was immunolocalized in ECs (D) but no visible immunolabeling was detected after base treatment (L); E and M. The CCRC-M34 epitope showing a weak immunolocalization in ECs (E) with no visible immunolabelling after base treatment (M); F and N. The JIM7 epitope immunolocalized in ECs (F), with weaker immunolabeling after HG de-esterification by base treatment (N); G and O. The LM20 epitope showing weak immunolocalization in ECs (G) which disappeared after base treatment (O); H and P. The LM5 epitope immunolocalized in starch-rich cells (arrows in H) showing less immunolabeling after base treatment (P). Bars represent 50 µm.

We compared immunolabeling patterns of pectin epitopes between ECs of cultivar ‘Yueyoukang 1’ and NECs of cultivar ‘Baxijiao’. Immunolabeling of the 2F4 epitope was weak in ECs (Fig. 5A), as it was in NECs (Fig. 4A). The JIM18 and LM5 epitopes were immunolabeled all over the section, in the cell walls of both surface and inner cell types of ECs (Fig. 5B, D) while the same epitopes appeared to be immunolabeled predominantly at the surface of NEC clumps (Fig. 4 B, D). Generally, immunolabelings of epitopes of highly methyl-esterified HG were relatively stronger in ECs (Fig. 5E–G) in comparison to NECs (Fig. 4E–G). The labeling of the LM5 epitope was relatively stronger in the starch-rich cells but it was weaker in the typical ECs (Fig. 5H). The response of pectic epitopes in ECs to the base treatment was generally very similar to that in NECs. After de-esterification with NaOH, immunolabeling of the 2F4 (Fig. 5I) and the LM18 (Fig. 5J) epitopes appeared to be relatively stronger. On the other hand, immunolabelings of JIM5, CCRC-M34 and LM20 epitopes became depleted (Fig. 5L, M, O), while they were weaker for JIM7 and LM5 epitopes (Fig. 5N, P).

### Distribution and methyl-esterification of pectin epitopes in pre-globular and globular embryos

**Figure 6:**
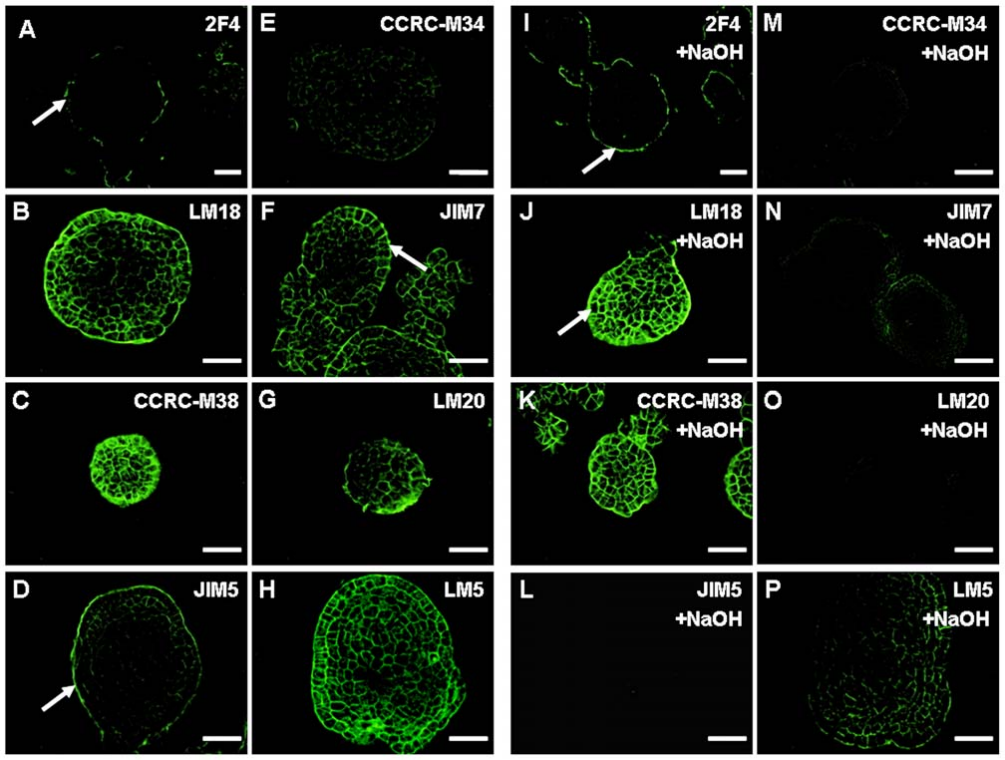
Immunofluorescence localization of pectin components in proembryos to early globular embryos of banana (*Musa* spp. AAA, cultivar ‘Yueyoukang 1’) and the effect of NaOH de-esterification on the immunofluorescence labeling of these epitopes. A and I. The 2F4 epitope was immunolocalized to the ECM at the surface of epidermal cells (arrow in A) while slight increase of immunolabeling was detected after base treatment (arrow in I); B and J. The LM18 epitope showing strong immunolocalization in epidermis and weaker in cortical cells (B) with increased immunolabeling after base treatment (arrow in J); C and K. The CCRC-M38 epitope showing strong and evenly distributed immunolocalization in the proembryo (C) with no visible changes after base treatment (K); D and L. The JIM5 epitope was immunolocalized in the ECM of epidermal cells (arrow in D) and this immunolabeling disappeared after base treatment (L); E and M. The CCRC-M34 epitope was weakly immunolocalized in the proembryo (E) and immunolabeling disappeared after base treatment (M); F and N. The JIM7 epitope was immunolocalized in the epidermis (arrow in F) and in the cortical cells (F), and these imunolabelings decreased after base treatment (N); G and O. The LM20 epitope showing moderate immunolabeling in the proembryo (G) but no visible immunolabeling after base treatment (O); H and P. The LM5 epitope showing evenly distributed immunolocalization in the proembryo (H) with immunolabeling decrease after base treatment (P). Bars represent 50 µm, except A and I where they represent 100 µm.

In pre-globular and globular embryos, the 2F4 epitope was detected by immunolabeling in the ECM layer surrounding these embryos (Fig. 6A). The JIM5 epitope was also strongly immunolabeled in this ECM layer while it also appeared in the subepidermal cells of such embryos (Fig. 6D). The CCRC-M38 epitope was immunofluorescently detected and equally distributed all over the pre-globular embryo (Fig. 6C). A comparable result was obtained also for the LM20 epitope (Fig. 6G). The LM18, JIM7 and LM5 epitopes were detected by immunofluorescence labeling in epidermis and subepidermis while they were hardly or not detectable in inner cells of globular embryos (Fig. 6B, F). Finally, the CCRC-M34 epitope was nearly undetectable by immunofluorescence labeling (Fig. 6E). As in previous cases, base treatments caused disappearance of the JIM5 and LM20 immunolabelings (Fig. 6L, O). Further, immunolabelings of the JIM7 and LM5 epitopes were largely reduced (Fig. 6N, P). On the other hand, immunolabelings of the 2F4 and LM18 epitopes appeared to be stronger after base treatment (Fig. 5I, J). Finally, no obvious difference in the immunolabeling intensity/distribution was detected for the CCRC-M38 epitope after NaOH treatment (Fig. 6K cf. 6C).

### Distribution and methyl-esterification of pectin epitopes in late-stage embryos

**Figure 7:**
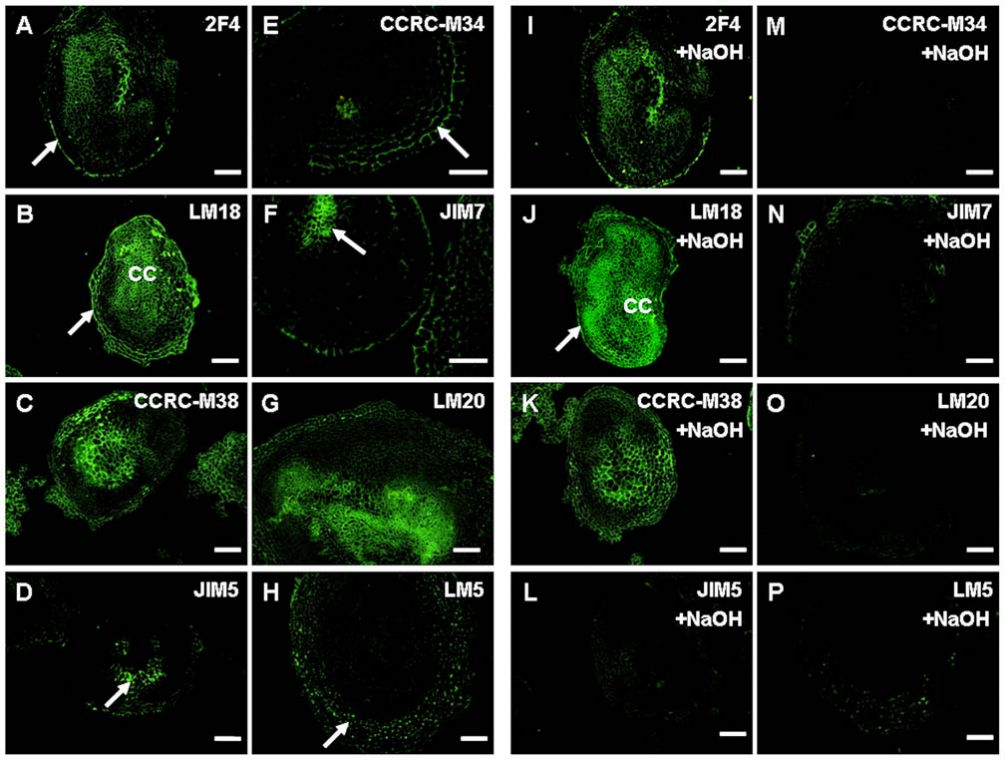
Immunofluorescence localization of pectin components in late embryos of banana (*Musa* spp. AAA, cultivar ‘Yueyoukang 1’) and the effect of NaOH de-esterification on the immunofluorescence labeling of these epitopes. A and I. The 2F4 epitope was immunolocalized in the ECM covering epidermal cells (arrow in A) but also in the cells in the middle parts of the embryos (A) with very slight increase of the immunolabeling after base treatment (I); B and J. The LM18 epitope was immunolocalized in epidermal (arrow in B) and cortical cells (B) with slight increase of the immunolabeling after base treatment (arrow in J); C and K. The CCRC-M38 epitope was immunolocalized to outer cell layers as well as to the cells in the middle part of embryos (C) with no changes after base treatment (K); D and L. The JIM5 epitope showing weak immunolocalization in epidermal and cortical cells but stronger one in parenchyma cells surrounding vascular tissue (arrow in D), and depletion of these immunolabelings after base treatment (L); E and M. The CCRC-M34 epitope showing weak immunolocalization in epidermal and cortical cells of late embryos (arrow in E) which disappeared after base treatment (M); F and N. The JIM7 epitope was immunolocalized to the cells of the middle part of late embryos (arrow in F), and this immuolabeling decreased after base treatment (N); G and O. The LM20 epitope showing relatively strong immunolocalization to the cells of the middle part of late embryos (G) with decrease of this immunolabeling after base treatment (O); H and P. The LM5 epitope was immunolocalized to cell-cell junctions (arrow) of cortical parenchyma cells (H), and this immunolabeling decreased after base treatment (P). Bars represent 50 µm in B, E, F and J; and 100 µm in other images.

Both 2F4 and LM18 epitopes were immunolocalized besides surface-localized ECM also to the cortical cells of the late-stage embryos (Fig. 7 A, B). The CCRC-M38 epitope was localized to outer cortical cells and to the parenchyma cells in the middle part of embryo (Fig. 7C) and the JIM5 epitope was localized especially in the cell-cell junctions of cortical cells (Fig. 7D). The CCRC-M34, JIM7 and LM20 epitopes were found in the epidermal cells and some inner parenchyma cells of late-stage embryos (Fig.6 E–G). The immunolocalization of LM5 epitope was restricted to the cell-cell junctions of cortical cells in these embryos (Fig. 7H). Base de-esterification of HG caused severe depletion of the immunolabeling in the case of JIM5, CCRC-M34 and LM2 epitopes (Fig. 7L, M, O). Moreover, also labelings of JIM7 and LM5 epitopes seemed to be reduced after the NaOH treatment (Fig. 7NP). The immunolabelings of 2F4, LM18 and CCRC-M38 epitopes were not obviously affected by base treatment (Fig. 7I–K). Altogether, these immunolabeling results suggested a tight developmental regulation of several pectic epitopes and their differential sensitivity to the NaOH de-esterification treatment. For better overview, a summary of immunofluorescence labeling results in control and NaOH-treated samples is presented in the Table 2.

**Table 2:** The intensity of immunolabeling with different pectin antibodies in diverse cell types during somatic embryogenesis of banana.

Tissue/organ	Cell type	2F4	CCRC-M38	JIM5	LM18	JIM7	LM2	CCRC-M34	LM5
NECs	Inner cells+NaOH	--	++++	--	-±	±-	±-	--	+++±
	Surface cells +NaOH	++±	++++++	++-	+++++++	±-	±-	--	+++±
ECSs	Typical EC+NaOH	--	++++	++-	+±++	+±	±-	±-	+-
	Starch-rich cells+NaOH	--	+++±	++-	+±++	+±	+±-	±-	++++
Pre and globularembryos	Epidermal cells+NaOH	++±	++++++	+±-	+++++	+±±	+±-	--	++++
	Inner cells+NaOH	--	++++++	±-	±++	±-	+	--	++±
Late embryos	Epidermal cells+NaOH	++±	++++	+-	++++	±±	±±	+-	--
	Sub-epidermal cells+NaOH	-±	++	--	--	--	--	+-	+±
	Cortical cells +NaOH	++±	++++	++-	+++++	+±	++±	--	--

Increasing intensity evaluated as - (no labeling);

± (very weak);

+(weak);

++ (middle);

+++ (strong).

NECs: non-embryogenic cells; ECs: embryogenic cells.

### The changes in the GalA content and pectin DM during somatic embryogenesis of banana

Comparison of D-galacturonic acid (GalA) content in different developmental stages during banana somatic embryogenesis revealed that it was the highest in the late-stage embryos (Table 3). The GalA content was significantly lower in NECs, ECs and pre-globular/globular proembryos, however, the differences among them were not significant (Table 3). On the contrary, DM was the lowest in the late-stage embryos and the highest in NECs. The moderate levels of DM were detected in ECs and pre-globular/globular embryos (Table 3).

**Table 3:** The degree of pectin methyl-esterification (DM) during somatic embryogenesis of banana.

	Non-embryogenic cells	Embryogenic cells	Pre-globular/globular embryos	Late embryos
Content of D-galacturonic acid (mg/g AIR)	101.26±4.95 b	100.57±2.23 b	97.50±4.55b	111.65±4.60 a
DM (%)	64.99	42.41	58.04	34.93

The data in the table represent an average of three biological replicates ± standard deviation (SD). Values marked by different letters (a, b) are significantly different by using Duncan's multiple range test at p<0.05.

AIR: alcohol insoluble residue; DM: degree of pectin methyl-esterification.

## Discussion

Pectins are major plant cell wall polysaccharides representing up to 35% of the primary cell wall in dicotyledonous plants and non-graminaceous (non-grass) monocots [11] while HG is the major pectin component. Biosynthesis and modifications of pectins, especially their methyl-esterification, have been proposed to be involved in plant development and cell adhesion [12], [13]. Moreover, biochemical properties of cell walls including content of galacturonan and uronic acid are variable according to taxonomy and distinctive cell walls occur in some groups of the monocotyledonous plant species [30]. Banana taxonomically belongs to the genus *Musa*, family *Musaceae* and order *Zingiberales* of monocotyledonous flowering plants. It has been reported that banana pericarp tissue contained high levels of both uronic acid and glacturonan [30]. Consistently with this report, we have found a relatively high content of the galacturonic acid in banana embryogenic cultures in this study. This indicates that some monocotyledonous plants contain pectic polysaccharides in the levels which are comparable to those in dicots [30].

Here we report on the developmental histochemical (ruthenium red) and immunofluorescence (using antibodies against diverse HG epitopes and the (1→4)-β-D-galactan epitope of RG-I) localizations of pectins along with developmental study on pectin DM during somatic embryogenesis of banana. Generally, all immunofluorescence data on intact cell walls should be interpreted with caution because immunolabeling of some pectic epitopes in certain cell walls/cell types might be blocked due to the sterical hindrance of these epitopes in these walls during immunolabeling procedure. We tried to partially avoid this problem by using several antibodies recognizing diverse epitopes at the same molecule, namely at pectic HG.

### Developmental localization of pectins in banana embryogenic cultures

Histochemical staining with ruthenium red revealed pink/red colored surface-localized pectins, especially at the surface of banana embryos at pre-globular, globular and pear-shaped developmental stages. This was in good correlation with immunolocalization patterns of surface-localized pectin epitopes (such as LM18, JIM5, JIM7 and LM20) at the same embryogenic developmental stages. Roughly, it was also consistent with immunodot labeling of these pectin epitopes during embryo development. Further, immunodot analysis correlated to immunolabeling with pectin antibodies suggested that banana embryogenic cultures were relatively rich in the JIM5 and LM5 epitopes, but poor in the 2F4 and CCRC-M34 epitopes. Among pectic epitopes, especially the highly methyl-esterified HG ones, recognized by LM20 and JIM7 antibodies, appeared to be developmentally regulated. Thus, both immunodot and immunofluorescence labelings of these two epitopes were weaker in embryogenic cells but stronger in pre-globular/globular and late embryos during embryo development. The immunolabeling intensities of other pectic epitopes on immunodots and immunofluorescence-labeled tissue sections also varied during embryo development, though the differences between diverse developmental stages were not as obvious as for LM20 and JIM7 epitopes.

This study revealed also some differences in pectins between nonembryogenic and embryogenic cultures of banana. For example, the LM5 epitope seemed to be more abundant in NECs as in ECs when immunodot and immunofluorescence labelings were compared to each other. This might be related to the fact that this epitope was mainly immunolocalized to the small groups of starch-rich cells in EC aggregates whereas it was detected in all cells in NEC aggregates. Moreover, clusters of NECs were covered by extracellular layer containing both LM18 and JIM5 epitopes. This might suggest extracellular secretion and local accumulation of these pectic epitopes in this ECM surface layer. On the other hand, the same epitopes were localized solely to the cell walls of individual ECs, suggesting that they were not secreted towards ECM surrounding ECs. Some of these results are in good agreement with previously published reports. For instance, larger intercellular spaces and the cell wall junctions contain JIM5 epitope in kiwifruit endosperm-derived callus [17]. The same epitope was found to be widely distributed over the middle lamella between adjacent meristematic cells of coconut calli while the JIM7 epitope was not so abundant and it was differentially distributed in cell walls [9]. Iwai et al. [27] reported that non-methyl-esterified pectins were abundant in carrot NECs while methyl-esterified ones were mostly detected in ECs of carrot. During development of globular somatic embryos of banana, the LM18 and JIM5 epitopes were detected in the outer surface of their epidermal cells. This was also the case for the LM18 but not for the JIM5 epitopes in the late-stage embryos. Moreover, globular and late-stage embryos possessed at their outer surfaces the 2F4 epitope, especially after NaOH treatment.

Previously, it was reported for monocot maize that highly methyl-esterified pectins recognized by JIM7 antibody were localized to the ECM covering embryogenic cell clumps of embryogenic callus [15]. Moreover, the JIM7 epitope was abundant in the outer wall of surface cells and the continuous layer over the cells in meristematic tissue of wheat representing another monocot species [19]. In banana, methyl-esterified JIM7 and JIM20 epitopes of HG were detected in epidermal/subepidermal cells of pre-globular and globular embryos and later also in the inner parenchyma cells of late-stage embryos.

### Is the 2F4 epitope of HG involved in cell-cell adhesion of banana ECs?

Formerly, acidic (low methyl-esterified) pectins were thought to play a crucial role in cell-cell adhesion [25], [31]. A minimum stretch of nine de-esterified GalA residues can form Ca^2+^ linkages, thus promoting the formation of so-called “egg-box” model structure [14], which may eventually strengthen the cell wall. The corresponding 2F4 epitope, however, likely does not play a major role in cell-cell adhesion in banana embryogenic cultures because it was weakly detected on both immunodots and tissue sections of tightly adhered ECs while it was more present in less-adhered NECs. In fact, this is in good agreement with published results that “egg-box” structures with characteristic calcium bridges were more abundant in carrot NECs as in ECs [27]. Additionally, the 2F4 epitope was not present in shoot apical meristem of *Sinapis alba* which is composed of tightly adhered cells [32]. Moreover, pectins in monocot wheat are suggested to play only a minor role in cell-cell adhesion [33]. Thus, it seems to be plausible that mechanism of intercellular attachment and cementing of cells is determined also by fine tuning of methyl-esterification degree of acidic pectins [25], [34] as well as by highly methyl-esterified pectins [32] in diverse plant species.

### Changes in degree of pectin methyl-esterification during somatic embryogenesis of banana

Modifications of HG such as methyl-esterification are important for cell fate determination and plant development [25]. Consequently, different levels of pectin methyl-esterification, as revealed by immunolabeling with JIM5 and JIM7 antibodies, have been reported for several plant species during somatic embryogenesis. In chicory, the JIM5 epitope was localized to the outer part of protodermal embryo cells and to the intercellular spaces while the JIM7 epitope was much less abundant during somatic embryogenesis [7]. Immunogold labeling with the JIM5 antibody revealed a high amount of low methyl-esterified pectins in the outer cell walls of *Citrus* proembryos [20]. Similarly, pectins recognized by the JIM5 antibody were rich in the peripheral wall below the exine and in the dividing inner cell walls of olive microspore-derived proembryos [10] as well as in the cells of cork oak proembryos [21]. In most of these studies immunolabeling of pectins with JIM7 antibody was very weak. Our immunodot and immunolocalization data with JIM5 and JIM7 antibodies on banana embryogenic cells and somatic embryos are consistent with above reports. In addition, we have used a much broader set of monoclonal antibodies binding to pectin HG epitopes with different degree of methyl-esterification from fully de-esterified (CCRC-M38) to highly methyl-esterified (LM20) in this study. This mapping of diverse pectic epitopes on immunodots and tissue sections suggests that fully de-esterified and low methyl-esterified ones such as CCRC-M38, LM18 and JIM5 were abundant in banana ECs while highly methyl-esterified ones such as CCRC-M34, JIM7 and LM20 antibodies were hardly detected. After ECs developed into embryos, the highly methyl-esterified pectins became more abundant.

### Conclusions and future prospects

This study provides new information about the developmental localization of cell wall pectins and about methyl-esterification patterns of pectin HG epitopes during banana somatic embryogenesis. The main conclusions are:

1. De-esterified and low methyl-esterified HG epitopes were detected in the surface localized ECM surrounding banana ECs and embryos.

2. Partially and highly methyl-esterified HG epitopes were more abundant in the cell walls of pre-globular and globular embryos.

3. De-esterification of pectins with base treatment caused imunolabeling depletion of highly methyl-esterified HG epitopes but increased immunolabeling of low methyl-esterified ones.

4. The data from the present study support the hypothesis that the mechanism of intercellular attachment and cementing of cells is likely determined not only by calcium pectate gels but also by certain acidic de-esterified pectins.

These results could be potentially valuable for development of future strategies aiming to improve the regeneration capacity of ECs in banana. This can be done, for example, by manipulation of pectin modifying enzymes such as pectin esterases and pectate lyases, as it was recently proposed for cyclamen somatic embryogenesis [35]. In the future, genetic manipulation of pectin modifying enzymes combined with mapping of pectin epitopes in transgenic plants might prove to be useful for biotechnological applications using somatic embryogenesis in banana.

## Materials and Methods

### Plant material

Embryogenic cells (ECs) of banana cultivar Yueyoukang 1 (*Musa* spp. AAA) as well as nonembryogenic cells (NECs) of cultivar Baxijiao (*Musa* spp. AAA) were cultured in ZZl medium [36], which is half-strength MS medium [37] supplemented with 1.1 mg/L 2, 4-dichlorophenoxyacetic acid (2, 4-D), 0.23 mg/L zeatin and 10 mg/L ascorbic acid. The pH of medium was adjusted to 6.0 before autoclaving. The cultures were incubated at 28±2°C under light on a reciprocal shaker at 90 rpm, and sub-cultured at 7 d interval. Seven days after the last subculture, ECs were inoculated on RD1 embryo-regeneration medium [36]. The cultures were incubated in the dark at 25±1°C and 29±1°C to promote development of somatic embryos.

### Histological staining of tissue sections with periodic acid-Schiff (PAS)

NECs, ECs (both at seven days after the last subculture), pre-globular/globular embryos (3-week-old regenerated material from the cultures incubated at 25±1°C) and late embryos (5-week-old regenerated material from the cultures incubated at 29±1°C) were collected and fixed with 3.7% (v/v) formaldehyde in stabilizing buffer MTSB [50 mM piperazine--N,N'-bis(2-ethanesulfonic acid (PIPES), 5 mM MgSO_4_.7H_2_O, 5 mM ethylene glycol-bis(2-aminoethylether)- *N,N,N',N'*-tetraacetic acid (EGTA), pH 6.9] at room temperature for 1 h. After washing in MTSB and phosphate-buffered saline (PBS) (pH 6.9), samples were dehydrated in graded ethanol series diluted in PBS and infiltrated with Steedman's wax according to previous studies [38]. Thin sections (8 µm) were mounted on microscopic slides coated with 0.2% polyethylenimine, de-waxed in absolute ethanol, re-hydrated in ethanol/PBS series and washed in PBS. Histological sections of NECs, ECs and somatic embryos were stained with PAS according to manufacturer instructions (Sigma).

### Staining of fresh material with ruthenium red

To test surface-localized pectins, suggesting an extracellular transport and accumulation of these pectins in mucilage-like ECMSN around NECs, ECs and somatic embryos at different developmental stages, the whole mount samples (without any fixation, embedding and sectioning) were collected at various developmental stages (see above) and incubated in 0.01% (w/v) ruthenium red by shaking (120 rpm) at 30°C for 2 h. Histochemical staining of surface pectins appeared as pink/red color and it was evaluated and documented under Leica binocular microscope (Leica, Germany).

### Antibodies and chemicals

All antibodies used in this study and their corresponding epitopes/antigens are presented in Table 1. These antibodies were obtained from PlantProbes (UK), except for CCRM antibodies which were obtained from Complex Carbohydrate Research Center (Athens, USA). All chemicals were of analytical reagent grade and were obtained from Sigma (St. Louis, USA) unless indicated otherwise.

### Immunofluorescence labeling

Immunolabeling was carried out exactly as described by Xu et al. [39], except for 2F4 antibody. For this antibody, the buffer containing 1 mM CaCl2 in 50 mM PIPES (pH 7.4, [40]) was used for sample fixation. Additionally, T/Ca/S buffer (20 mM Tris-HCl, 0.5 mM CaCl2, 150 mM NaCl, pH 8.2, [41]) was used instead of PBS before labeling with the secondary antibody during immunolabeling procedure. The secondary antibody for 2F4, CCRC-M34 and CCRC-M38 primary antibodies was anti-mouse IgG-FITC (F9006, Sigma), and for all JIM and LM antibodies it was anti-rat IgG-FITC (F6258, Sigma), respectively. Sections incubated only with secondary antibodies were used as negative controls. A minimum three slides were used for each antibody at each developmental stage. Fluorescence was examined and documented with an Olympus BH-2-FRCA microscope.

### Preparation of alcohol-insoluble residue (AIR)

AIR was prepared according to the method described by Louvet et al. [42]. In brief, samples were washed three times with 70% ethanol (v/v) at 70°C for 30 min after homogenization. The supernatant was removed after centrifugation. The pellet was crushed in liquid nitrogen and freeze-dried.

### Immunodot assay

Pectin was extracted from AIR with 0.5% (w/v) ammonium oxalate buffer at 100°C and the concentration was adjusted to 1 mg/ml. Samples were spotted (as 5 µl drops) onto nitrocellulose membrane by a micropipette. The membrane with dots was air dried at room temperature for 1 h. Assays with JIM5, JIM7, LM5, LM18, JIM20, CCRC-M34 and CCRC-M38 antibodies were carried out as described by Willats et al. [43]. T/Ca/S buffer was used to replace PBS for the assay with 2F4 antibody. After the final wash, the membrane was developed in 3, 3′-diaminobenzidine tetrahydrochloride (DAB) kit from TCI (Shanghai) Development Co., Ltd.

### Measurement of pectin methyl-esterification degree (DM) with colorimetry

The DM was calculated as moles of methanol per mol of GalA. The methanol assay was adopted from the methods described by Klavons and Bennett [44] and Louvet et al. [42]. In detail, 5 mg of AIR was saponified in 2 ml of 0.25 M KOH at room temperature for 1 hr, then neutralized with phosphoric acid (to pH 7.5). After centrifugation at 10.000 g for 10 min, aliquots of the supernatant (1 ml) were loaded into a 15 ml tube. Alcohol oxidase (1 ml, 1 U/ml, diluted in distilled water, Sigma) was added to each tube. After gently mixing, the tube was incubated at room temperature for 20 min. Thereafter, 2 ml of a mixture containing 0.1% 2,4-pentanedione in 1 M ammonium acetate and 0.14% acetic acid was added. Following 15 min of incubation at 60°C, samples were cooled on ice and absorbance was measured at 420 nm (HAITACHI, U-2900).

Methanol in potassium phosphate buffer (pH 7.5) (0–20 µg/ml range) was used as a standard. GalA content of the sample was determined colorimetrically by the metahydroxydiphenyl assay adopted from Blumenkrantz and Asboe-Hansen [45] and from Louvet et al. [42]. In detail, 5 mg of AIR was hydrolyzed in 125 µl of 13 M sulfuric acid at room temperature for 30 min. A second hydrolysis was performed. The sample was diluted 5 times with distilled water and saponified at a final concentration of 1 M NaOH at 100°C for 2 h. The supernatant was adjusted to pH 8 with NaOH followed by neutralization with HCl. After centrifugation at 10.000 g for 10 min, aliquots of the supernatant (0.5 ml) were loaded into a 15 ml tube. Pre-cooled samples (on ice-water bath) were supplemented with 1.5 ml of pre-cooled 0.025 M sodium tetraborate buffer in concentrated sulfuric acid and incubated at 100°C for 5 min. After cooling in a water-ice bath, 25 µl of 0.15% MHDP in 0.5% NaOH was added to the samples. Following 10 min incubation at room temperature, absorbance was recorded at 520 nm. GalA (0–100 mg/l range) was used as a standard. Three replicates were made for each treatment and each experiment was repeated twice. GalA was calculated as micrograms of GalA per gram of AIR.

### Chemical de-esterification

The extensive degradation of pectic polymers was performed by base catalyzed de-esterification. Samples were prepared as described above for immunofluorescence labeling. After dewaxing, sample sections were treated with 0.05 M NaOH at 4°C for 30 min followed by modified immunolabeling protocol (washing with buffer two times for 10 min, blocking with glycine).
